# Bypass Grafting for Myocardial Infarction From Endocarditis Embolus to the Left Anterior Descending

**DOI:** 10.1016/j.atssr.2024.04.027

**Published:** 2024-05-23

**Authors:** Rohun Bhagat, Milind Desai, Shinya Unai

**Affiliations:** 1 Department of Thoracic and Cardiovascular Surgery, Cleveland Clinic, Cleveland, Ohio; 2 Department of Cardiovascular Medicine, Cleveland Clinic, Cleveland, Ohio

## Abstract

A 67-year-old man with aortic valve endocarditis presented with acute ST-segment elevation myocardial infarction and was found to have embolic vegetation occlusion of the left anterior descending coronary artery. This patient was successfully treated with early aortic valve replacement, extraction of a vegetation embolus, and coronary artery bypass grafting over the site of extraction.

This case report highlights the complexity of managing acute ST-segment elevation myocardial infarction (STEMI) resulting from an embolic vegetation from aortic valve infective endocarditis. Additionally, we describe a surgical revascularization and concomitant aortic valve replacement (AVR) approach to management of this presentation.

A 67-year-old man with aortic valve regurgitation caused by aortic valve endocarditis presented for a scheduled preoperative workup. He had new-onset, persistent chest pain on awakening that morning. His past medical history included asthma, hypertension, coronavirus disease-2019, and syncope (attributed to dehydration).

This patient had a 6-month history of intermittent fevers and a 30-pound weight loss after a root canal procedure. In early 2023, this patient was admitted for fever and generalized weakness to an outside hospital, where he was given a diagnosis of infective endocarditis caused by *Streptococcus mitis*. A transesophageal echocardiogram showed an aortic valve vegetation measuring 1.7 × 0.4 cm and moderate aortic valve insufficiency. Computed tomography showed evidence of focal infrarenal aortic dissection extending into the left common iliac artery with an unclear cause and a possible small left renal infarct. During this previous admission, the patient denied chest pain, dyspnea, and shortness of breath. The outside hospital cardiac surgery team offered this patient surgical AVR with antibiotics vs discharge on intravenous antibiotics for a total of 4 to 6 weeks with an interval echocardiogram and blood cultures at 4 to 6 weeks after discharge. The patient opted for intravenous antibiotic therapy without AVR at that time. The patient was discharged on a 4-week dose of intravenous ceftriaxone with plans for repeat blood cultures and an echocardiogram near the end of antibiotic treatment. The results of follow-up blood cultures were negative.

The next month, the patient presented to the outside hospital with acute-onset right lower quadrant pain. Repeat computed tomography showed infrarenal abdominal aortic dissection similar to that found in the previous admission with wedge-shaped hypoattenuation of the right kidney likely representing an acute to subacute infarct. The patient’s presentation and symptoms for this admission were thought to be related to an aortic valve embolic phenomenon causing a renal infarct. A transthoracic echocardiogram at this time showed persistent vegetation on the aortic valve with mild to moderate aortic insufficiency. The patient was offered surgery but opted to seek a second opinion at our hospital (Cleveland Clinic, Cleveland, OH).

The patient presented to the Cleveland Clinic for preoperative evaluation. At this time, he was asymptomatic. Routine cardiac catheterization showed moderate diffuse coronary artery disease with severe ostial stenosis of the first septal perforator and of a small, short obtuse marginal artery. Of note, the left anterior descending (LAD) coronary artery had moderate diffuse disease with no specific area of concern.

The next day, on the morning of his cardiac surgery preoperative visit, the patient awoke with new-onset chest pain. He presented for his routine visit that day and he noted this chest pain. An electrocardiogram was obtained, which showed concern for an anterolateral STEMI (Figure 1) with an associated troponin level of 800 ng/L (subsequently rising to 1400 ng/L). The patient was taken for urgent left-sided heart catheterization, which showed 90% narrowing of the distal LAD artery that had not been seen on routine catheterization the previous day (Figure 2).

**Figure 1:**
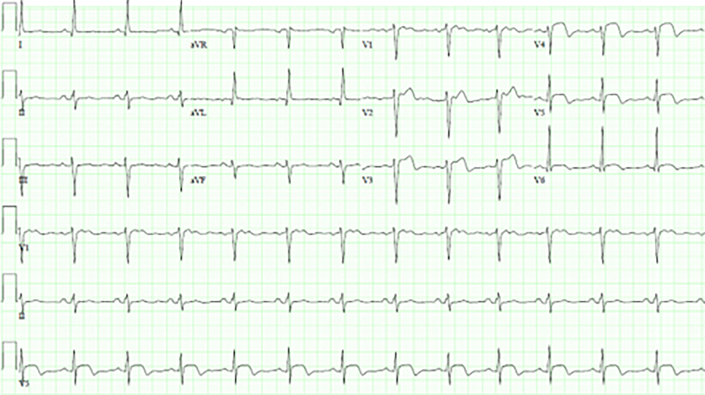
Electrocardiogram concerning for acute anterolateral ST-segment elevation myocardial infarction.

**Figure 2:**
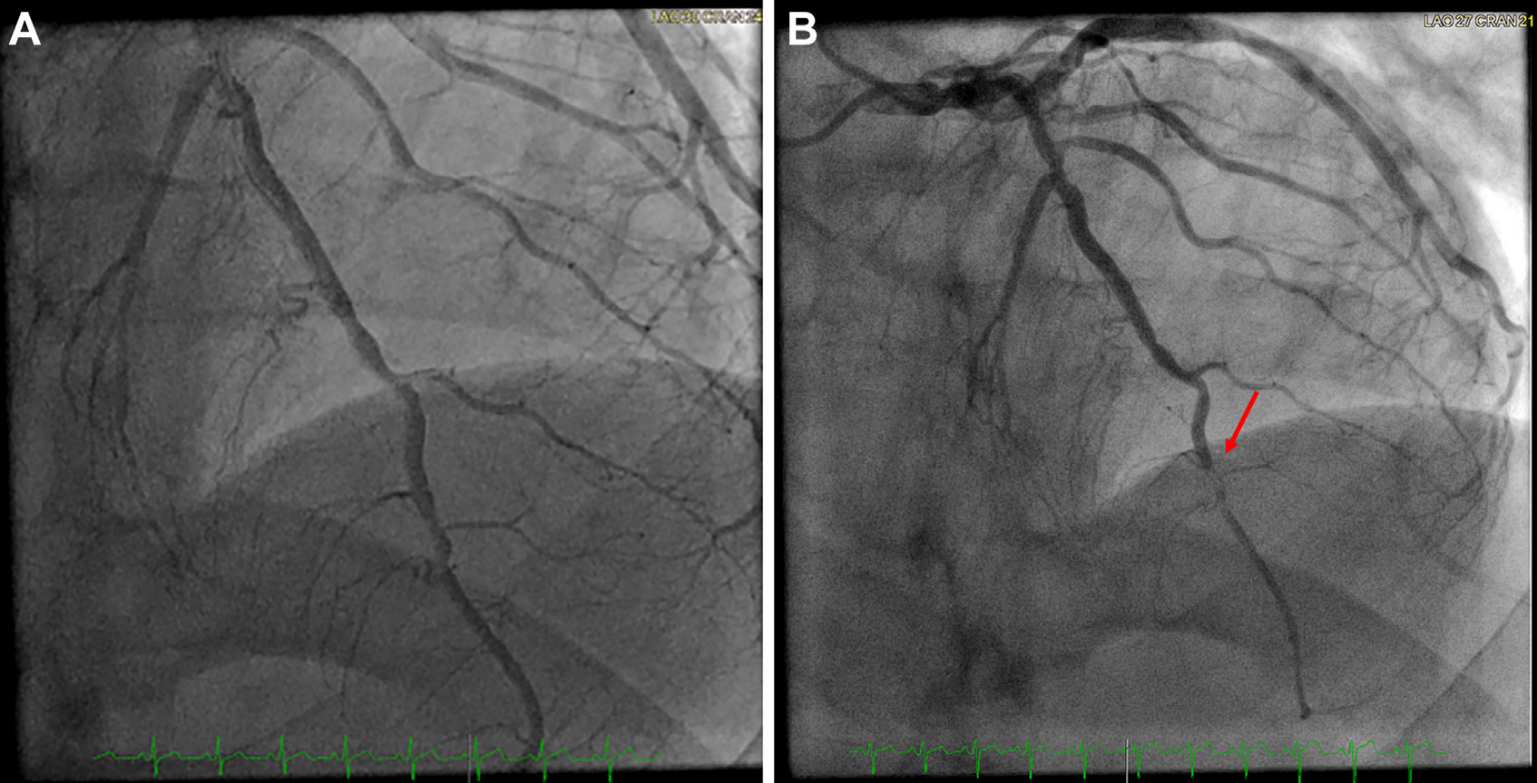
(A) Left-sided heart catheterization from the day before the acute ST-segment elevation myocardial infarction (STEMI) presentation. (B) Left-sided heart catheterization from the day of the acute STEMI presentation. The arrow indicates the new lesion within the left anterior descending coronary artery.

At the time of left-sided heart catheterization for new-onset chest pain, the LAD artery lesion was thought to be distal, with a relatively small-caliber vessel distal to the occlusion. Coronary artery revascularization by percutaneous coronary intervention was deferred. The surgical team was informed, and the patient was admitted for medical treatment (heparin and vasodilator therapy) preceding the AVR planned for the next morning.

The patient was brought to the operating room the next morning for AVR. A preoperative transesophageal echocardiogram did not show any wall motion abnormalities. At the time of incision, coronary artery bypass grafting (CABG) was not part of the operative plan. On direct inspection of the heart and coronary arteries, the distal LAD artery was thought to be of greater caliber than appreciated on angiography. A suitable conduit was harvested for LAD artery bypass. Once cardiopulmonary bypass was initiated and the heart was arrested, attention was turned to the acutely occluded portion of the LAD artery. The artery was dissected from the surrounding epicardial fat and incised, revealing an intraarterial embolus (Figures 3A, 3B). The embolus was successfully extracted from the artery (Figures 3C, 3D), and a bypass was performed with the distal target being the site of the extracted embolus.

**Figure 3:**
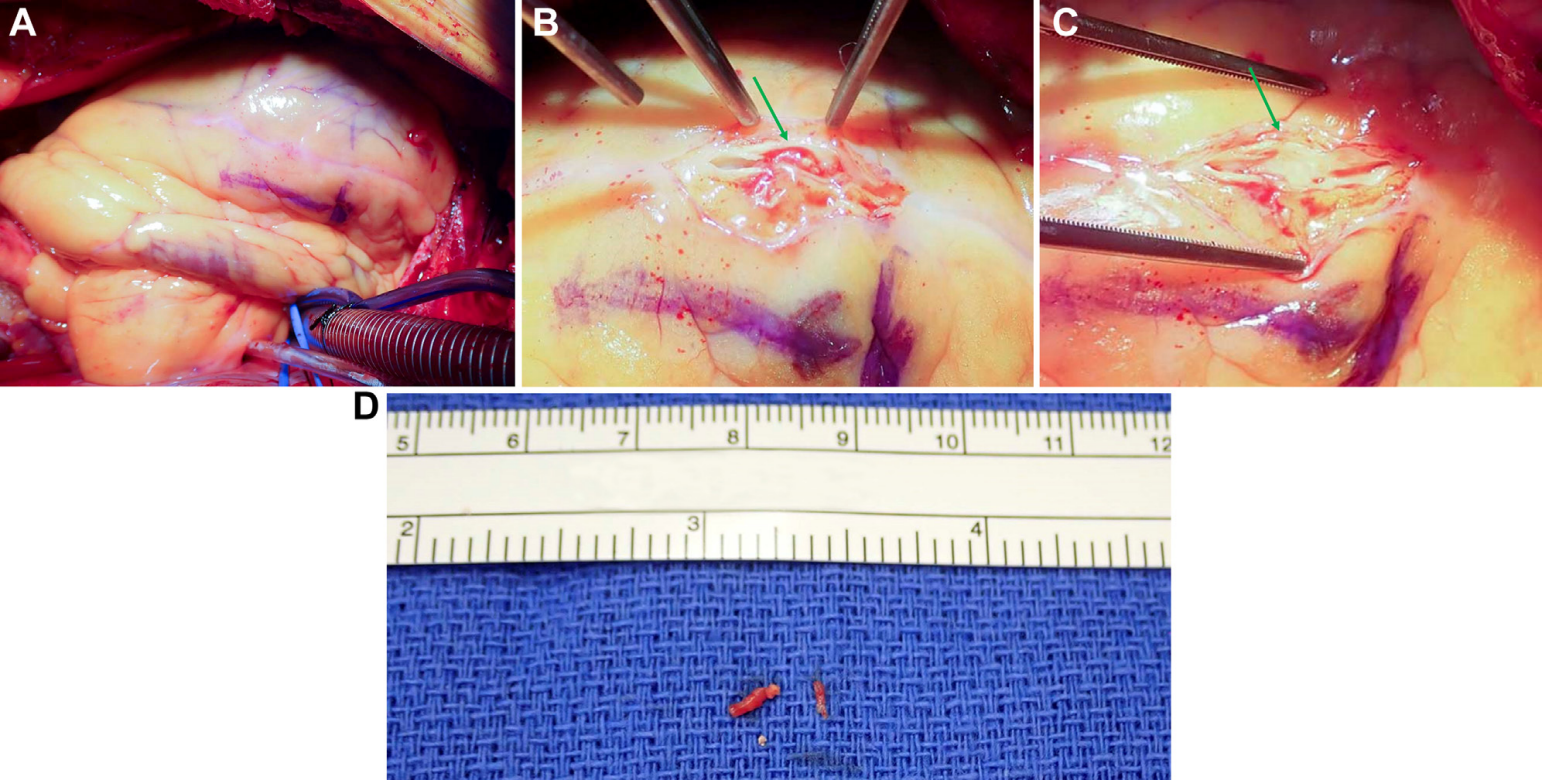
(A) Cannulated heart with a marker noting the inferior border of the region of interest of the left anterior descending coronary artery. (B) Incised left anterior descending coronary artery showing the embolic vegetation within the lumen (arrow points to the embolic vegetation). (C) Incised left anterior descending coronary artery after extraction of the embolic vegetation from the lumen (arrow points to the embolus-free lumen of the left anterior descending coronary artery). (D) Extracted embolic vegetation.

The aortic valve was trileaflet with vegetations, consistent with infective endocarditis. This valve was uneventfully replaced with a tissue bioprosthesis.

## Comment

STEMI resulting from embolic aortic valve infective endocarditis has been rarely described in the literature. In 9 previous published cases, 3 of the 9 patients had anterior territory myocardial infarctions, 2 of the 9 had anterolateral territory infarctions, and 4 of the 9 had inferior territory infarctions.^1^ Treatment of these previous cases of acute STEMI in the setting of aortic valve infective endocarditis varied. Specifically, 3 of the 9 patients underwent angioplasty (2 of these 3 patients survived), 3 of the 9 patients underwent AVR with or without CABG (2 of these 3 patients survived, and 1 of the 3 did not report survival status), 1 of the 9 patients received fibrinolytic therapy and survived (with a complication of a major gastrointestinal bleeding episode), and 2 of the 9 patients neither received fibrinolytic therapy nor underwent angioplasty (1 of the 2 survived).^1^ Overall, excluding 1 case that did not report survival status, 6 of 8 patients survived beyond the immediate observation period.^1^

We describe a 10th case of embolic aortic valve infective endocarditis manifesting as an acute STEMI with an anterolateral territory infarction from occlusion of the LAD artery. This patient was successfully treated with surgical AVR, extraction of a vegetation embolus, and bypass grafting of the LAD artery.

Acute STEMI resulting from an embolic vegetation from aortic valve infective endocarditis is a rare, complex presentation with no guideline-directed management. Although a routine acute STEMI is often treated with angioplasty, an embolic vegetation presents a novel, significant factor when considering treatment modality. As an initial concern, placement of a foreign material directly in contact with a vegetation theoretically places the foreign material at high risk for seeding and infection of the material. Additionally, unlike progressive coronary artery disease and plaque rupture, an embolic vegetation has the potential to cause a mycotic aneurysm in the associated coronary artery, a complication that has been described in the literature.^2^

Although it is understandable that a hemodynamically unstable patient may require urgent angioplasty, it is worth considering the possibility of deferring angioplasty in a stable patient in favor of surgical extraction of the embolic vegetation followed by bypass of the affected vessel, especially if the patient requires surgical valve replacement. Specifically, extraction of the embolic vegetation may be instrumental in avoiding complications such as stent infection or mycotic aneurysm development.

The patient was brought to the cardiac surgery intensive care unit after the operation. The patient recovered well and was ultimately discharged without complications on postoperative day 5 on 4 weeks of intravenous antibiotics. The final pathology report of the extracted embolus showed an acute thrombus with a calcified vegetation containing bacterial cocci, consistent with an infective endocarditis embolus.

The patient was asymptomatic and recovering well 3 months postoperatively.

An acute STEMI presentation in the setting of aortic valve endocarditis with imaging-proven coronary artery embolus may be successfully treated with early surgical AVR, surgical extraction of a vegetation embolus, and CABG over the site of extraction.

## References

[1] Peddi K., Hsu A.L., Ayala T.H. (2019). Infective aortic valve endocarditis causing embolic consecutive ST-elevation myocardial infarctions. Case Rep Cardiol.

[2] Singh M., Mishra A., Kaluski E. (2015). Acute ST-elevation myocardial infarction due to septic embolism: a case report and review of management options. Catheter Cardiovasc Interv.

